# Multiple-Choice Item Distractor Development Using Topic Modeling Approaches

**DOI:** 10.3389/fpsyg.2019.00825

**Published:** 2019-04-25

**Authors:** Jinnie Shin, Qi Guo, Mark J. Gierl

**Affiliations:** Centre for Research in Applied Measurement and Evaluation, Department of Educational Psychology, University of Alberta, Edmonton, AB, Canada

**Keywords:** multiple-choice items, distractors, misconceptions, distractor generation, latent dirichlet allocation

## Abstract

Writing a high-quality, multiple-choice test item is a complex process. Creating plausible but incorrect options for each item poses significant challenges for the content specialist because this task is often undertaken without implementing a systematic method. In the current study, we describe and demonstrate a systematic method for creating plausible but incorrect options, also called distractors, based on students’ misconceptions. These misconceptions are extracted from the labeled written responses. One thousand five hundred and fifteen written responses from an existing constructed-response item in Biology from Grade 10 students were used to demonstrate the method. Using a topic modeling procedure commonly used with machine learning and natural language processing called latent dirichlet allocation, 22 plausible misconceptions from students’ written responses were identified and used to produce a list of plausible distractors based on students’ responses. These distractors, in turn, were used as part of new multiple-choice items. Implications for item development are discussed.

## Introduction

Multiple-choice testing is one of the most enduring and successful forms of educational assessment that remains in practice today. Multiple-choice items are used in educational testing because they permit the measurement of diverse types of knowledge, skills, and competencies (Haladyna, 2004; Downing, 2006; Popham, 2008). Multiple-choice items are efficient to administer; they are easy to score objectively; they can be used to sample a wide range of content; they require a relatively short time to administer (Haladyna, 2004; Haladyna and Rodriguez, 2013; Rodriguez, 2016). Downing (2006, p. 288), in his seminal chapter in the *Handbook of Test Development*, claimed that selected-response items, like multiple choice, are the most appropriate item format for measuring cognitive achievement or ability, especially higher-order cognitive skills, such as problem solving, synthesis, and evaluation. He also stated that this item format is both useful and appropriate for creating exams intended to measure a broad range of knowledge, ability, or cognitive skills across many domains.

Because of these important benefits, multiple-choice items continue to have broad appeal and, hence, application in education, despite some potential disadvantages, such as guessing effects and unintentionally exposing students’ to wrong information. North American students take 100s of multiple-choice tests and answer 1000s of multiple-choice items as part of their educational experience. Chingos (2012) reported that one-third of the United States use multiple-choice items exclusively for assessing 4th grade and 8th grade students’ math and reading skills. In higher education, a multiple-choice test is a common and widely used assessment format for measuring students’ knowledge, especially in introductory courses with a large group of students. Multiple-choice testing is also used extensively for international assessments. In the 2015 administration of The Trends in International Mathematics and Science Study (TIMSS), for example, half of the mathematics and science items used the multiple-choice format (Mullis et al., 2016). In the 2015 administration of the Program for International Student Assessment (PISA), two-third of the items in reading, mathematics, and science assessments were multiple choice (OECD, 2016).

A multiple-choice item consists of a stem, options, and auxiliary information. The stem contains context, content, and/or the question the student is required to answer. The options include a set of alternative answers with one correct option and one or more incorrect options or distractors. Auxiliary information includes any additional content, in either the stem or option, required to create an item, including text, images, tables, graphs, diagrams, audio, and/or video. To answer a multiple-choice item, the student is presented with a stem and two or more options that differ in their relative correctness. Students are required to make a distinction among response options, several of which may be partially correct, in order to select the best or most correct option. Hence, the student must use her or his knowledge and problem-solving skills to identify the relationship between the content in the stem and the correct option. The incorrect options are called distractors because they are considered to be “distracting” to students with partial knowledge due to their plausibility to yield the correct option.

Creating multiple-choice items is a challenging task, particular when it comes to distractor development, because of the sheer volume of work that is required. For example, to create 100 multiple-choice items that consists of one correct option and four incorrect options, a content specialist has to create 100 stems and 100 correct options. The content specialist also needs to create 400 plausible but incorrect options. This challenge of distractor development is both daunting and, oftentimes, unsuccessful. Haladyna and Downing (1993) evaluated the distractors from four standardized multiple-choice tests. They evaluated the quality and plausibility of distractors based on the attractiveness of distractors. More specifically, they emphasized that plausible distractors should be able to attract more than 5% of the low-performing students, who failed to identify a correct answer. Based on such criteria, they found that only 8% of the items contained effective distractors.

To overcome the challenge of creating large numbers of effective distractors, researchers and practitioners have explored and implemented different strategies. The most common strategy focuses on a list of plausible but incorrect alternatives linked to common misconceptions or errors in thinking, reasoning, and problem solving (Haladyna and Downing, 1989; Case and Swanson, 2001; Vacc et al., 2001; Collins, 2006; Moreno et al., 2006, 2015; de la Torre, 2009; Tarrant et al., 2009; Rodriguez, 2011, 2016). Haladyna and Rodriguez (2013) in their textbook *Developing and Validity Test Items* claim that the most effective way to develop plausible distractors using misconceptions is to identify “common errors” elicited by a particular stem in the item prompt. These common errors serve as candidates for plausible distractors. Haladyna and Rodriguez state that common errors can be identified in two ways. First, they can be identified using the judgments of contents specialists who have a good understanding of teaching and learning within a specific content area and who can specify the common errors and misconceptions that arise when students learn a new topic or concept. Second, they can be identified by evaluating student answers to constructed-response item (i.e., an item that contains a stem by no options) where errors in reasoning, thinking, and problem solving are documented in the student’s responses. The second approach—extracting student responses from constructed-response items—is the preferred strategy for identifying common errors because it is based on the actual response processes from students rather than the expected response processes inferred from the judgment of content specialists about how students respond to test items. However, identifying and extracting common errors and misconceptions from the actual response processes is a daunting task because large amounts of response data must be processes and this data, in turn, must be classified accurately in order to identify outcomes that could be used as distractors.

The purpose of this study is to introduce an augmented intelligence approach for systematically identifying and classifying misconceptions from the students’ written responses that are pre-labeled for the purpose of creating distractors that can be used for multiple-choice items. Augmented intelligence is an area within artificial intelligence that deals with how computer systems can emulate and extend human cognitive abilities thereby helping to improve human task performance and to enhance human problem solving (Zheng et al., 2017). It requires the interaction between a human and a computer system in order for the system to produce an output or solution. Augmented intelligence combines the human capacity for judgment with the ability of modern computing using computational analysis and data storage to solve complex and, typically, unstructured problems. Augmented intelligence can therefore be used to characterize any process or system that improves the human capacity for solving complex problems by relying on a partnership between a human and a machine (Pan, 2016; Popenici and Kerr, 2017).

We introduce and demonstrate an augmented intelligence method that can be used for distractor development using latent dirichlet allocation (LDA; Blei et al., 2003). LDA is a statistical model used in machine learning and natural language processing which identifies specific topics and concepts within written texts. Specific words are expected to appear in a written text more or less frequently given a particular topic. LDA can be used to capture this expected outcome in a mathematical framework by focusing on the number of times words appeared in written text for different topics. Using LDA, content specialists can identify actual misconceptions based on students’ response processes in order to create lists of plausible distractors.

### Traditional Approach for Distractor Development

Distractors are one of the key components that affect the overall quality of multiple-choice items as well as the item’s statistical characteristics (Gierl et al., 2017). Distractors are intended to distinguish between students who have not yet acquired the knowledge necessary to answer the item correctly from those who understand the content. Therefore, distractors in a multiple-choice item are designed to contain plausible but incorrect answers based on students’ common errors or misconceptions so that the option can measure students’ level of mastery in a specific content area (e.g., Case and Swanson, 2001; Ascalon et al., 2007; Hoshino, 2013; Towns, 2014; Lai et al., 2016). Creating distractors using common errors and misconceptions result in multiple-choice items with increased diagnostic value as well as higher item quality (Haladyna and Downing, 1989; Case and Swanson, 2001; Briggs et al., 2006; Moreno et al., 2006, 2015; de la Torre, 2009; Tarrant et al., 2009; Rodriguez, 2011, 2016).

Haladyna and Rodriguez (2013) claimed that common errors and misconceptions could be identified using two different approaches. In the first approach, content specialists create individual distractors by hand that contain these common errors and misconceptions. Collins (2006) recommended that content specialists mimic students’ problem solving processes by answering questions such as, “what is a common error for solving this problem?” and “what do students usually confuse this concept or idea with?” in order to identify plausible distractors. The most appealing aspect of this method lies in its practicality and ease of implementation. The distractors are created by content specialists familiar with the students and the content area to mimic the typical and the commons problems that are most likely to occur. While this approach is feasible, it is also based on three assumptions. First, plausible algorithms, rules, or sources of information can be specified by content specialists. Second, plausible but incorrect distractors can be produced using these sources. Third, the misconceptions identified by the content specialists from these sources are, in fact, the same misconceptions held by the students. Proper alignment of the assumptions is critical for creating distractors that measure students’ actual errors and misconceptions. Moreover, the alignment must occur for each distractor across every multiple-choice item. Using our earlier example, if a content specialist writes 100 multiple-choice items and each item contains five options (i.e., one correct option and four distractors), then the content specialist must identify 400 plausible but incorrect alternatives that satisfy these three assumptions.

In the second approach, students’ responses from existing constructed-response items are evaluated to identify common errors and misconceptions. That is, content specialists review students’ responses from constructed-response items to identify mistakes, errors, and misunderstanding and then classify these outcomes to create a compiled list of plausible distractors (e.g., Bekkink et al., 2016). This approach addressed the inferential problem associated with the previous approach because it is based on actual student response data rather than judgments about expected response processes. In other words, approach two is data driven. Common errors and misconceptions identified using approach two come from the algorithms, rules, or sources of information used by students to produce incorrect answers. Unfortunately, the second approach suffers from the problem of practicality and ease of implementation because it is neither practical nor easy to use. As it is currently implemented, approach two is daunting because it entails a comprehensive review of students’ written responses using a manual process with the goal of identify common errors and misconceptions that occur consistently and systematically. It is also a process fraught with interpretive problems because identifying common errors and misconceptions that occur systematically can be a subjective task (e.g., what are the characteristics of a systematic misconception). And, despite the potential benefits of using a data-driven approach, practically also dictates that the item development process should be relatively quick and efficient, even when large number of multiple-choice items are required. This requirement is challenging to address using the second approach, especially when large amounts of written text are available from a constructed-response item.

To-date, limited research has been conducted to investigate the application of augmented intelligence for the purpose of distractor development. Researchers have explored the significance of using students’ misconceptions and common errors to create distractors. The approach used in these studies was based on identifying misconceptions using students written or verbal responses that, in turn, were manually categorize by content specialists to identify common errors and misconceptions (e.g., Vacc et al., 2001; Haladyna and Rodriguez, 2013; Moreno et al., 2015; Bekkink et al., 2016; Rodriguez, 2016). As noted earlier, a data-drive approach using students’ responses is inherently beneficial for identifying the actual errors and misconceptions that students use when they produce incorrect answers. But it is also inherently limited because it is excessively time consuming and labor intensive to identify and classify errors from written text using a manual review process. To overcome this limitation, we introduce and illustrate a data-driven method for creating distractors based on student’s common errors and misconceptions using LDA.

### Topic Modeling and Latent Dirichlet Allocation

Locating keywords and topics to understand text is a simple and effective way for humans to classify textual information. To gather information about certain topics, for example, we often start from generating one or two key words to locate relevant documents that share common topics. Unfortunately, this approach quickly becomes unmanageable for humans when the amount of textual information begins to increase. For example, having content specialists manually review 1000s of students’ responses to identify and then categorize common errors would be a time consuming and inefficient classification exercise.

To overcome this clustering challenge, topic modeling has been developed and used with machine learning and natural language processing algorithms to uncover the hidden topics in a document (Blei, 2012). These hidden topics can be identified without any pre-labeling, which means that topic models do not require pre-categorized or topic-labeled documents. In machine learning, these problems are described as an unsupervised learning approach, which means the structure of the problem includes targets or outputs which are unknown and hence the primary focus of learning is to understand the structure of the data. Therefore, in topic modeling, we attempt to identify hidden or unobserved target, topics, using the fully observed information, words.

If we assume that a sequence of words in a document is governed by the same unobserved topic, then we could simply compute the likelihood of a document to represent certain topic to determine the underlying topic of a document in an unsupervised setting. To find the common topics, topic modeling uses word occurrence information where certain words are expected to appear in a document more or less frequently depending on a particular topic. LDA is a generative probabilistic topic modeling algorithm (Blei et al., 2003), where each document is perceived as a mixture of several topics. Generative models take the information of how observed data was generated into account to build a model. Suppose, for instance, we have documents that were generated by complex procedures that are unknown.

**FIGURE 1:**
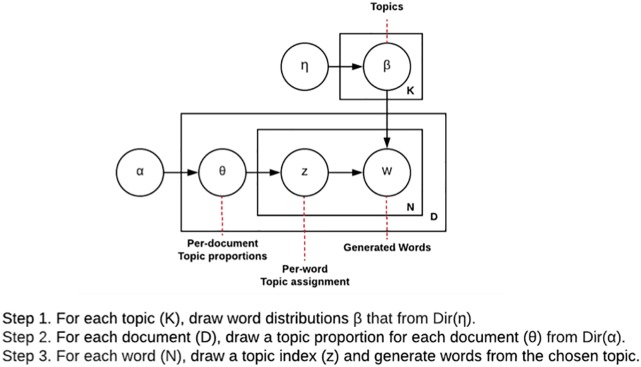
A conceptual representation of latent dirichlet allocation (LDA).

Latent dirichlet allocation attempts to synthesize an approximated generation procedure and observed information (i.e., words) to uncover hidden topics, without any labels. Moreover, unlike other topic modeling approaches, LDA can not only produce interpretable topics and can handle unseen documents to assign topics. The generative process of LDA consists of three layers of sampling a topic distribution, sampling topics, and sampling words over topics. For example, after the number of words (or document length) and the number of topics are decided, a topic distribution is specified (e.g., 40% biology, 30% kinetics, and 30% psychology). Next, a topic is picked based on the topic mixture distribution and a word is picked based on the distribution over words corresponding to the topic. This process is then repeated until all the words are generated for each documents. Figure 1 describes a graphical representation of the generative process of LDA.

Given this process, LDA attempts to explore the hidden topics in a document by computing a posterior distribution of the hidden variables given a document. Due to a large number of possible topic structures, computing the probability of certain words under a specific topic (i.e., the distribution over words corresponding to the topic) becomes impossible to compute. To address this problem, LDA uses a method called Gibbs sampling (Porteous et al., 2008) where each word is randomly assigned in the document to one of the topics, which will provide the initial guess of the word-topic and word-document distribution. LDA assumes that all topic assignments except for the current word in question are correct, and then updates the assignment of the current word. This process is repeated to improve the assignment until a steady state is reached. Once the final assignment is identified, it is used to estimate the topic mixtures of each document.

### Model Evaluation and Augmented Intelligence

While topic models can be used to extract meaningful and interpretable topic assignments, evaluating the final assignment is challenging using an unsupervised approach (Chang et al., 2009). Unsupervised learning tasks do not include pre-labeled targets. Instead human judgment is required to evaluate the practicality and usefulness of the topic modeling performance (Konrad, 2017). For example, the practicality of the topic model could be evaluated using the “human-in-the-loop” augmented intelligence approach, where humans are asked to locate a randomly substituted word or topic (Chang et al., 2009). If the human can reliably tell which one is a random intruder, then we can say that the trained topic yields a coherent and discernible topic (Chang et al., 2009). In addition, intrinsic measures (i.e., statistical measures) should also be considered for model evaluation. Such measures help evaluate how well the model fits the observed data.

Log-likelihood evaluates the probability of the observed data, given the model (Griffiths and Steyvers, 2004). Thus, we can locate the best model by attempting to produce the highest log-likelihood measure. The Kullback-Leibler (KL) divergence measure focuses on measuring the divergence among the topic distributions. KL divergence explicitly focuses on evaluating how much information we lose when we choose a certain model, by computing the symmetric KL divergence between the distribution of variance in the topic-word distribution and the marginal topic distribution (Cao et al., 2008; Arun et al., 2010). Thus, the best model can be determined by locating the point where the KL divergence measure reaches the lowest value (Arun et al., 2010).

Previous research has been conducted to demonstrate the usefulness of LDA for different types of topic modeling assignments. In education, for example, LDA has been used to uncover topics for essay scoring purposes (Meisner, 2018), implementing course recommendation systems (Apaza et al., 2014), and evaluating teachers (Moretti et al., 2015). However, to our knowledge, LDA has never been used to identify students’ errors and misconceptions for the purpose of creating distractors that could be used to create multiple-choice items. Therefore, the purpose of the study is to describe a method for creating distractor by identifying students’ misconceptions using the LDA topic modeling approach. Unlike the traditional approach where content specialists were responsible for using their judgments to analyze and evaluate students’ responses in order to identify plausible misconceptions for distractors development, the current study provided a systematic and data-driven method to cluster students’ written responses with similar underlying concepts in order to locate common mistakes. Once clustered, these responses become the basis for creating plausible distractors.

## Materials and Methods

### Data

An open source data set collected and released from the short-answer scoring competition called Automated Student Assessment Prize (ASAP) was used in the study ^1^. As the data set is publicly available, ethical approval was not sought in the study. ASAP was held in 2012. The competition was designed to promote the capabilities of effective scoring system using automated essay scoring frameworks and to provide efficient classroom essay scoring tools for practitioners. The competition included two phases. The first phase focused on developing robust automated scoring frameworks for relatively long responses (up to 650 words). The second phase focused on scoring short responses (up to 50 words). Both the competitions significantly contributed to promoting open and rigors model development for automated essay scoring (Shermis, 2014, 2015).

For the short-essay scoring competition, 10 data sets were released and each data set was generated from a single prompt. The responses were produced by students in grade 10. Each data set was based on a unique prompt in different disciplines, such as Language Arts, Biology, and Science. All the responses were pre-labeled, scored by two human-raters. The current study used data set six from Biology to demonstrate the proposed method. This data was chosen to demonstrate the proposed method for three reasons. Fist, the current method requires pre-labeled data set and the data set six consisted of the resolved-score (or final score) based on the agreement of the two human raters. Second, the prompt required students to respond using multiple answers thereby producing a variety of diverse responses from a single prompt. In addition, the original constructed-response prompt could be easily reformatted into a multiple-choice stem.

More specifically, we used 1,515 responses from the original training set, where students were asked to list and describe three processes used by cells to control the movement of substances across the cell membrane (see Appendix A). The particular number of training responses were selected based on the score assigned by two independent human raters. The final score corresponded to the number of correctly identified answer and we only selected the responses where students failed to identify any correct answer (i.e., score 0), as the focus of this study is on extracting common errors and misconceptions.

#### Distractor Development Stage 1: Data Preparation

To achieve clear and interpretable clusters of topics, pre-processing is required. First, all of the misspelled words were corrected. Second, words were converted into lower cases and lemmatized using the Python NLTK library (Bird et al., 2009). Lemmatization is the process of grouping the words together so they can be analyzed as a single item based on their dictionary form. For example, the words ‘studies’ and ‘studying’ would be lemmatized into ‘study.’ Third, digits, non-alphabetic words (e.g., #, %, &, @), and stop words (e.g., a, and, but, how) were removed and all punctuation was specified as a separate word. Fourth, responses were separated into sentences allowing each sentence to be denoted as a separate topic.

Pre-processing is also focused on spelling correction using a combination of several approaches. We used the word embedding-based model for spelling correction. Word embedding-based models use the semantic similarities of words to determine the best candidate of a misspelled word (Nagata et al., 2017, see Appendix C). We used a list of words provided in the pre-trained GloVe embedding (Pennington et al., 2014), which were trained on six billion words from Wikipedia 2014 and Gigaword 5. We attempted to locate the best candidate of an incorrect word from the Glove embedding word list based on a cosine-similarity score. Using the embedding-based spell correction, we could successfully correct more than 95% of the misspelled words, while some of the remaining misspelled words that could not be fixed with the methods were correctly manually. This approach was chosen after attempting existing spell checkers in Python and the correction results were relatively lower than expected (e.g., NLTK edit-distance with 78% correction). Such cases often included words that were significantly malformed, thus, providing very limited resemblance with a correct form.

#### Distractor Development Stage 2: Topic Clustering and Cluster Evaluation

The LDA model was constructed using the Python library lda 1.0.5. To generate clear and interpretable clusters of topics, model training and evaluation took place simultaneously. To enable flexible and robust learning, it is necessary to identify the ranges of several model parameters so the model with the optimum range can be identified. For example, the number of topic groups must be specified before training begins. The number of Gibbs sampling iteration must also be specified to train the model. To begin, the number of topics and sample iterations ranged from 1 to 50 and up to 800 iterations, respectively. These ranges were selected so that we can extract as many potential misconceptions as possible with a stable estimation. We set our initial range of the number of topics as a relatively large number, 50, so that the model could conduct a comprehensive categorization of common errors and misconceptions. In terms of the number of iterations, we evaluated the negative log-likelihood of the model at every 10 iterations and inspected whether a significant decrease or increase in log-likelihood occurred. The significance was evaluated based on a chosen tolerance value of 0.5. The results indicated that log-likelihood stabilized around 800 iterations. The performance of our initial model was evaluated using the perplexity measure. Perplexity is a commonly used topic-model measure that is computed by dividing a negative log-likelihood by the number of words (see Appendix C). As the name suggests, perplexity provides the degree of ‘uncertainty’ or ‘confusion’ the model has in assigning probabilities to text. Therefore, we could determine the optimal number of topics by locating the model with the lowest perplexity.

Then, the topic clusters were visualized to evaluate the clustering. Topic clusters were projected in a two-dimensional space by computing the distance between topics using t-distributed stochastic neighbor embedding (t-SNE). T-SNE is a dimensionality reduction algorithm for high-dimensional data visualization. The idea of t-SNE is to find a probability distribution that is a function of the smallest number of coordinates and to create a similar distribution function to reduce the dimensionality. Assume that we want to calculate the probability of finding two points i and j at the squared Euclidean distance between the points, ||*x_i_* − *x_j_*||^2^. T-SNE attempts to match the distribution using a Student’s-*t* distribution, while attempting to learn the *y* coordinates of the points (i.e., *y_i_* and *y_j_*) in the lower dimension. If the visualized clusters are significantly overlapping and malformed, then the number of topics should be adjusted. In addition, the KL divergence was used as an evaluation criterion for the visualization because it helps determine the similarity of the two distributions. The learning algorithm attempts to create a clear visualization of distinctive topic clusters while minimizing KL divergence to locate the optimal model. To do so, several adjustments were necessary to determine the number of iterations, the learning rate, and the perplexity rate. While the number of iterations and the learning rate determines the efficiency and accuracy of model learning through controlling for the weight adjustments, the perplexity rate controls for the effective number of cluster neighbors. Finally, interpretability of the clusters was evaluated by summarizing the clustered sentences using the Python library genism summarization. Gensim summarization conducts a text rank-based summarization using a variation of the TextRank algorithm (Barrios et al., 2016). TextRank attempts to construct a graph from a document, where sentences (or nodes) are connected with each other via edges. Edges represent the similarity between the sentences, which are often computed based on the word overlap between the two sentences. TextRank hypothesizes that the most important sentence in a text as the one that is the most frequently connected in a graph. We chose this approach as previous studies have demonstrated relatively good performance using the method, while it does not require any manual annotation (Mihalcea and Tarau, 2004). The summaries were created so that content specialists could effectively evaluate the plausibility of the extracted common errors and misconceptions.

In the study, we refer to content specialists as the experts who are experienced in item writing in particular subjects. With this type of content expertise, validating the plausibility of summarized common errors and misconceptions could improve the quality of distractors which are generated from each topic cluster. To do so, content specialists could discuss and attempt to identify where each misconception originated from. For example, if the content of a cluster includes morphologically or phonetically similar words with correct answers, the specialists could conclude that the misconception originated from the confusion in recalling certain terminologies or associating a term with a correct definition. Also, content specialists could be encouraged to answer more concrete questions to evaluate the quality of clusters. Such questions could include, “How many of the clusters do you find meaningful?” and “Is the cluster describing a commonly well-identified misconception regarding the topic?” This would help content specialists to evaluate distractors thoroughly, while providing important information to evaluate the capacity of the current system.

#### Distractor Development Stage 3: Item and Distractor Formation

In stage 3, content specialists formulate distractors using the common errors and misconception clusters identified in the previous stage. We propose several methods that could promote more systematic distractor development using students’ misconceptions. The distractor generation process can be distinguished based on the question type (or stem) that content specialists pose regarding a topic. First, the content specialists could decide to change the format of the original question from the constructed-response item to a multiple choice item format, while attempting to measure the same construct of interest (e.g., which of the following procedures is correct about cell movement?). In this case, we could use the cluster summarizations and the key words and phrases directly. In stage 2, we explored how each misconception cluster can be represented using key words and summarization. Thus, using key words or summarized sentences as distractors would be able to attract students with different levels of understanding effectively. Alternatively, content specialists could develop a question that focuses on specific sub-concepts of a topic. Active- or passive-transport could be good examples of sub-concepts to evaluate, that is closely associated with the original question. In this case, distractors could be directly located based on students’ responses from the cluster, where students appeared to have trouble understanding the concepts of active- and passive-transport. We will present how the two methods can be utilized more thoroughly using examples in the next section.

Generating distractors using students’ misconceptions have been identified as one of the most effective way in developing multiple-choice items (Haladyna and Rodriguez, 2013). However, with our augmented intelligence approach, which require content specialists’ judgment in the evolution process, we believe the effectiveness of distractors could still significantly depend on the content specialists judgments. Therefore, while we encourage further studies on the effectiveness of the distractors generated using the proposed methods, it was out of our scope of research to provide empirical results on behaviors of distractors in a real test setting. We will discuss such concerns more thoroughly in the limitation section with several suggestions for future research.

## Results

### Topic Clustering and Cluster Evaluation Results

In the original constructed-response item, students were asked to provide three correct responses to the following item: “List and describe three processes used by cells to control the movement of substances across the cell membrane.” The results indicated that the optimal LDA model identified 22 common misconceptions. The number of topic clusters were selected based on the log-likelihood measure as well as the KL divergence. The model achieved a perplexity of 34.76 after 800 iterations and the lowest KL divergence of 40.50 with 22 topics. As discussed earlier, the log-likelihood measure provides the probability of the observed data given the model (Griffiths and Steyvers, 2004).

In addition, the interpretability and plausibility of each topic cluster was evaluated using extracted key words and summaries. A full list of topic key words and summaries can be found in Appendix B. Six to eight topic key words were used for each topic cluster. They were chosen based on the strength of association to represent the topic cluster and the strength was measured by weights assigned to each word. In addition, summaries were generated for each cluster to increase their interpretability. This information was designed to help the content specialists to interpret students’ common errors and misconceptions and to evaluate the representativeness of the clusters to form plausible distractors. For example, topic 20 included several key words, such as ‘mRNA,’ ‘RNA,’ ‘tRNA,’ ‘DNA,’ ‘information,’ ‘translation,’ ‘transcription,’ and ‘messages.’ Content specialists formed their initial impression on each misconception based on these key words. In addition, by reading the summary which states “mRNA carries messages from the nucleus to other organs tRNA transports DNA to places with in the cell rRNA,” content specialists can understand specific contexts and associations among the key words more thoroughly so they can make more informed decision about whether the cluster could be used to create a plausible distractor which represents a common error or misconception.

### Item and Distractor Formation Results

A set of distractors were generated using the evaluated clusters of students’ common errors and misconceptions. In addition to create distractors for the originally proposed item, where students were required to describe three processes used by cells to control the movement of substances across the cell membrane, we explored the capacity of the current method in generating distractors on additional cluster-specific items. The following examples introduce a step-by-step breakdown of the distractor generation procedures.

#### Example 1: Generating Distractors for the Original Prompt

**FIGURE 2:**
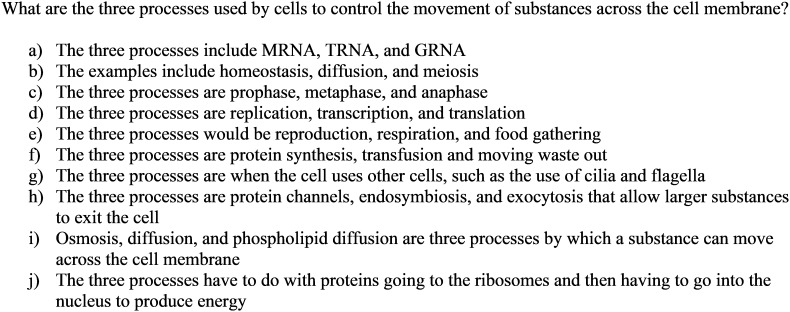
An example question and distractors generated for the original prompt.

As shown in Figure 2, a multiple-choice item was created from the original constructed-response item. Reflecting the original prompt, the stem was changed to “What are the three processes used by cells to control the movement of substance across the cell membrane?” To generate distractors that could each reflect different common error and misconception, the list of options was created by locating students’ responses with key words from the stem, such as ‘processes,’ ‘movement,’ or ‘substances’ from each misconception topic cluster. More specifically, the option *g* represents the cluster 13 (see Appendix B), where students describe the movement of flagellum as part of the movement of substances across the cell membrane. In this example, the correct answer is i, while the other options were produced to represent students’ misconceptions.

#### Example 2: Generating Distractors Using Additional Prompts

**FIGURE 3:**
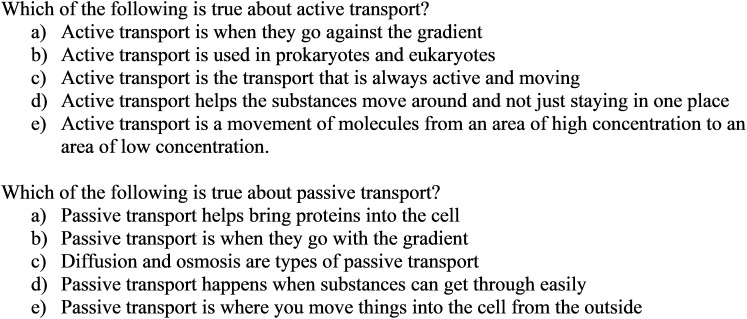
Example questions and distractors generated for the sub-topics of the original prompt.

As shown in Figure 3, the proposed method could be extended to generate distractors for cluster-specific items. Cluster-specific items refer to items that are generated to further evaluate students’ understanding that reflect the misconceptions captured in a particular content cluster. For example, Figure 3 introduces two cluster-specific items, which were posed based on students’ responses in cluster 2 (see Appendix B). In cluster 2, students had trouble correctly explaining and distinguishing between the two concepts of active and passive transports. Therefore, to evaluate students’ understanding on active and passive transport, two additional multiple-choice stems were created: “Which of the following is true about active transport?” and “Which of the following is true about the passive transport?” To generate distractors for the cluster-specific items, we implemented the same process where the key words and phrases (i.e., active transport, passive transport) were used to locate students’ responses that included these key terms. Unlike the first example, the distractors were only located among the responses in cluster 2 as the items were created based on cluster 2. The correct option is *a* and *b*, respectively.

## Discussion

**FIGURE 4:**
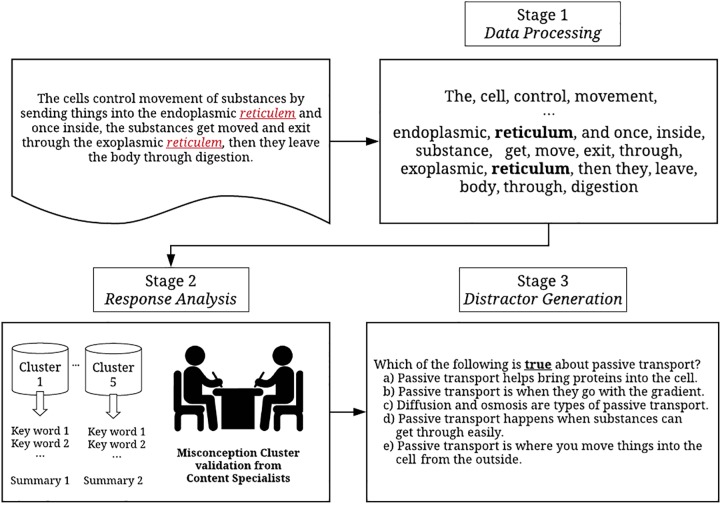
A comprehensive framework of the distractor generation process.

The recent introduction of different applications of augmented intelligence in educational assessment have brought about dramatic changes in the field by promoting efficient new test development and administration procedures (Popenici and Kerr, 2017). Augmented intelligence, which is a branch of artificial intelligence, helps content experts broaden their capabilities and make more informed decision in a timely manner with appropriate technological support. For instance, with a machine-aided scoring system, experts can score essays more efficiently because the machine can be used to help distinguish problematic essays that fail to map onto a scoring rubric from more coherent essays. Currently, little research has been conducted to investigate the application of augmented intelligence in item development, especially as it relates to creating distractors. Effective distractors can attract students with a partial understanding, in other words, discriminating students who have not yet reached the mastery level of comprehension regarding the concept. Thus, generating effective distractors is directly associated with increasing the quality of an item and its characteristics (i.e., item difficulty and discrimination; DiBattista and Kurzawa, 2011). Studies have been conducted to explore the significance of using students’ misconceptions and common errors to create distractors (e.g., Vacc et al., 2001; Moreno et al., 2015; Rodriguez, 2016). Misconceptions are typically gathered using students written or verbal responses on similar or connected topics and content experts manually categorize and identify plausible misconceptions using the written response evidence (Bekkink et al., 2016). In other cases, content experts attempt to mimic students’ thought processes in order to identify plausible errors (Haladyna and Rodriguez, 2013). However, these approaches are unfeasible when large numbers of items must be created. To overcome this limitation, we introduced and illustrated a data-driven method for generating distractors based on misconceptions from students’ written responses using the workflow presented in Figure 4.

It is important to acknowledge that the current methods attempt to incorporate both machine- or data-driven and experts-driven approaches harmoniously in every stage. While the data-driven approach provides prominent benefits in facilitating a systematic and effective distractor generation process, we believe the intervention from experts could help improving the system, behaving as a gatekeeper for quality insurance of the final product, distractors. Especially in educational assessments, content experts’ decisions are often considered a reference or gold-standard in making the ultimate high-stakes decisions. The steps in Figure 4 workflow were used to identify 22 distinct clusters of common errors and misconceptions using students’ written responses from a constructed-response item in Biology. In the first data processing stage, we primarily used the data-driven approach to pre-process the responses (e.g., lemmatization, tokenization, remove punctuations, and non-alphabetic words). Also, while we corrected the majority of misspelled words using the embedding-based approach, it was still required to conduct a few manual corrections. In the response analysis stage, clusters were created automatically using a topic-modeling approach, then, content experts were required to evaluate the interpretability and plausibility of the extracted clusters, the information was used to generate a list of 22 plausible distractors that, in turn, helped create a parallel multiple-choice item. A parallel multiple-choice item refers to an item originally presented as a constructed-response task that has been reformatted into a selective-response task. The quality of generated distractors can be further empirically evaluated by pilot testing in a classroom evaluation setting and we will discuss more details about the evaluation of item characteristics in the next section.

### Implications for Future Research

The current study has implications for distractor writing practices, specifically, and item development, more generally. Topic modeling allows content experts to use student responses in a more adaptive and productive way. Written responses represent an enormous source of valuable information about students’ understanding, which is not only related to the construct of interest, but also to misconceptions about that construct. To-date, little effort has been spent exploring the use of machine learning methods for gathering and using information about misconceptions that can be found is students constructed responses. Using the method described and illustrated in this study, researchers and practitioners can now use the written responses gathered in assignments and tests to plan future lessons and to create more student-adapted learning activities and assessments. The method can also be used to provide evidence for students’ developmental level of understanding about certain concepts. For example, by analyzing the responses from the higher-ability group and compare the misconception clusters with the ones from the lower-ability group, more in-depth information can be gathered to create a comprehensive picture of how students’ level of understanding develops on specific concepts and within specific content areas.

### Distractor Development and Item Generation

Potentially the most important future application of this method resides in its application to automatic item generation (AIG; Irvine and Kyllonen, 2002; Gierl and Haladyna, 2013). AIG is a relatively new but rapidly evolving research area where cognitive and psychometric modeling practices guide the production of tests that include items generated with the aid of computer technology. Gierl and Lai (2013, 2016) developed a three-step process for AIG. In step 1, content specialists create a cognitive model for AIG.

Currently, distractor development poses a unique and consequential problem in AIG in the step 2 item modeling stage. For the selected-response format, items must not only include a stem with a corresponding correct option, but also include a set of distractors. Distractors in AIG are typically designed from a list of plausible but incorrect alternatives linked to misconceptions identified by content specialists. Because AIG produces 100s of items, strategies are needed to create a correspondingly large number of plausible but erroneous distractors. Distractor development for AIG is now guided by the distractor pool method with random selection (Gierl and Lai, 2016). To identify the content for the distractors, content specialists identify a list of plausible but incorrect options that are appropriate for all possible items generated with a given item model. Then, distractors are randomly selected from this pool of plausible but erroneous content and added to each generated item. This method is based on the assumption that a pool of plausible distractors can be created. A sample of these plausible distractors are selected at random to complete the item generation process. The strength of this method is its simplicity. This method can yield large numbers of distractors. The weakness of this method resides with the strong assumption that all pooled distractors are equally plausible and appropriate for all generated items. Equal plausibility and appropriateness is strong and, in many cases, restrictive assumption. Also, there is little reasoning to guide how distractors are paired with the correct option because pairing is achieved with random assignment.

To improve the plausibility and appropriateness of the distractors, rules, and rationales that yield errors or misconceptions can be used to create distractors. Distractor rationales are short descriptions that specify the reasoning which underlies each option. These rationales are currently provided by content specialists. But the rules can also be created using the method presented in our study to produce distractors that conform to specific, empirically-based, student misconceptions. Hence, distractors can be created systematically so that each distractor matches a rationale. This proposed approach could be called the *systematic generation with rationales method*. It would be based on the assumption that algorithms, rules, and procedures can first be articulated by content specialists and then used to create plausible but incorrect alternatives linked to students’ actual misconceptions or errors in thinking, reasoning, and problem-solving. The strength of this method is that the distractors are much more specific and, hence, plausible and appropriate, especially when compared to the distractor pool method with random assignment. Hence, integrating the outcomes from the topic modeling methods presented in this paper with new developments in AIG should be considered an important area of future research.

### Limitation and Future Research

Even though the study was carefully designed and structured to minimize potential error with results and further interpretations, we found the three key limitations that should be addressed and carefully considered for future research: the main purposes of our study were to introduce a novel method of identifying students’ misconceptions in a systematic manner to encourage efficient distractor generation for multiple-choice item development. Thus, our study could not investigate the item behaviors with generated distractors in a real test setting. Investigating the item behaviors in relation to the distractor quality would help us further understand the importance of item development with well-performing distractors. For example, DiBattista and Kurzawa (2011) demonstrated how the plausibility of distractors significantly affects item characteristics (e.g., item discrimination) in classroom assessment. Therefore, we encourage future researchers to evaluate the plausibility and effectiveness of the generated distractors to explore the significance of our proposed method thoroughly. Second, our current method required labeled responses to identify students’ responses with incorrect answers. Scoring students’ responses manually can be a very expensive and tedious procedure, especially in a large-scale assessment. However, as the current method attempts to extract students’ misconceptions that could be located from their incorrect responses, it is necessary to score or use pre-labeled data set to properly implement the proposed method. This could somewhat limit the usability of the proposed method as locating domain specific and pre-labeled data can be a daunting challenge. However, we believe such limitations can be readily overcome by using automated essay scoring systems (see Appendix C) to generate labeled responses in advanced to implement the current method. Last, augmented intelligence approach of our method aim to create a systematic method to distractor development supporting content experts to make informed decisions using misconception clusters. Therefore, it is important to investigate whether content specialists, indeed, feel supported to make informed decisions in creating distractors. We encourage future research to carefully evaluate the affective factors of content experts in using this method to fully evaluate the capacity of the current method.

## Author Contributions

JS, QG, and MG contributed in conceptualization and formalization of research ideas of the study. JS located and organized the data. JS and QG performed the analysis. JS and MG wrote the first draft of the manuscript. All authors contributed to manuscript revision, read and approved the submitted version.

## Conflict of Interest Statement

The authors declare that the research was conducted in the absence of any commercial or financial relationships that could be construed as a potential conflict of interest.

## Footnotes

1 www.kaggle.com/c/asap-sas

## Appendix A

**Prompt—cell membrane item**

List and describe three processes used by cells to control the movement of substances across the cell membrane.

**Rubric for cell membrane**

Key elements:

• Selective permeability is used by the cell membrane to allow certain substances to move across.
• Passive transport occurs when substances move from an area of higher concentration to an area of lower concentration.
• Osmosis is the diffusion of water across the cell membrane.
• Facilitated diffusion occurs when the membrane controls the pathway for a particle to enter or leave a cell.
• Active transport occurs when a cell uses energy to move a substance across the cell membrane, and/or a substance moves from an area of low to high concentration, or against the concentration gradient.
• Pumps are used to move charged particles like sodium and potassium ions through membranes using energy and carrier proteins.
• Membrane-assisted transport occurs when the membrane of the vesicle fuses with the cell membrane forcing large molecules out of the cell as in exocytosis.
• Membrane-assisted transport occurs when molecules are engulfed by the cell membrane as in endocytosis.
• Membrane-assisted transport occurs when vesicles are formed around large molecules as in phagocytosis.
• Membrane-assisted transport occurs when vesicles are formed around liquid droplets as in pinocytosis.
• Protein channels or channel proteins allow for the movement of specific molecules or substances into or out of the cell.

Rubric:

3 points

Three key elements

2 points

Two key elements

1 point

One key element

0 points

Other.

## Appendix B

**Table:** Representative key words of topic clusters

Topic	Key words	Summary
1	Cell, osmosis, water, diffusion, membrane, process, permeable, moving	Three processes used by cells to control the movement of substances across the cell membrane are being selectively or semi permeable, osmosis, and diffusion
2	Transport, active, diffusion, passive, osmosis, processes, facilitated, type	Three types of controlled movement of substances across the cell membrane include passive transport, active transport, and diffusion
3	Cell, substance, membrane, way, moves, cytoplasm, goes, organism	Another one is where the organism extends out sections of its cell membrane and fills it with cytoplasm while the opposite end goes away and it moves by a crawling type movement
4	Cells, blood, body, make, flow, need, brain, send	The movements of substances across the cell membrane flow through the blood streams
5	Cell, membrane, wall, help, nucleus, things, outside, inside	Three processes used by cells to control the movement of substances across the cell membrane is flagella which helps the cell get through the membrane, the nucleus that is the control center, and the cell wall to protect the cell from any unwanted cells or anything unwanted
6	Cell, waste, food, gets, stuff, nutrients, needed, needs	The Golgi bodies help by getting rid of stuff not needed in the cell
7	Protein, proteins, cell, enzymes, synthesis, channel	The cell uses three basic processes for movement across the membrane one is the flagellum, another cytoplasm and finally the protein in the ribonuclease acid
8	Cell, membrane, movement, control, substances, helps, plasma, different	Pores in the membrane allow substances in and out of the cell and Golgi body helps transport substances in and out of cell
9	Cells, use, proteins, way, membrane, ribosomes, carry, proteins	Cells use vesicles, transport chains, and proteins to control the movement of substances across the cell membrane
10	Cell, substance, membrane, diffusion, concentration, substances, movement, uses	Osmosis is the movement of water going from a low concentration to a high concentration in the cell membrane
11	Golgi, nucleus, proteins, apparatus, ribosomes, reticulum, endoplasmic, use	The ribosomes produce the energy for the cell the Golgi apparatus gets rid of waste and the nucleus hold all the information and DNA
12	Cell, things, wall, membrane, inside, getting, substances, lets	Cell wall makes the plant cell stiff but also keeps out unwanted items or organism’s cell membrane lets things in and out of the cell with permission from the nucleus and chloroplast help the plants maintain energy
13	Like, flagellum, flagella, use, cilia, cell, helps, help	One way of movement is the use of flagellum which is a long tail like structure the moves behind the cell
14	Cells, substances, use, process, place, organelles, moving, help	Another processes but which cells use to control the substances that cross the cell membranes are the phospholipids that line that cell wall these help keep unwanted thing out as well
15	Movement, cells, control, used, cell, processes, substances, membrane	Three processes that cells use to control the movement of substances across the cell membrane is protein synthesis, transfusion, and moving waste out
16	Cells, mitosis, meiosis process, reproduce, make, makes, meiosis	Another processes but which cells use to control the substances that cross the cell membranes are the phospholipids that line that cell wall these help keep unwanted thing out as well
17	Cell, controls, membrane, nucleus, goes, wall, tells, comes	The cell uses three processes by the names of meiosis, mitosis, and cell reproduction
18	Cell, uses, energy, things, membrane, moves, mitochondria, endocytosis	The nucleus controls everything and the mitochondria tell what enters and leaves the cell
19	Respiration, cellular, reproduction, photosynthesis, process, food, division, homeostasis	Endocytosis which is part of active transport, where the cell uses energy to pull items through the selectively permeable membrane
20	mRNA, tRNA, RNA, DNA, information, translation, transcription, messages	It does this to maintain homeostasis the cell moves oxygen in and carbon dioxide of out of the cell through the process of cellular respiration
21	Cell, membrane, certain, let, things, substances, enter, allow	mRNA carries messages from the nucleus to other organs tRNA transports DNA to places with in the cell rRNA
22	Anaphase, telophase, thing, prophase, metaphase, second, interphase, know	Three of the processes that cells use to control movement into and out of the cell membrane are protein channels that let substances pass through them, endosymbiosis allows large substances to enter, and exocytosis allow larger substances to exit the cell
