# Supplementary material for: Multiple-Choice Item Distractor Development Using Topic Modeling Approaches
Source: Front Psychol. 2019 Apr 25;10:825. doi: 10.3389/fpsyg.2019.00825 (PMC6524712; doi:10.3389/fpsyg.2019.00825)
Supplement: Supplementary file 1 [file Data_Sheet_1.pdf]

Appendix C

KEY TERMS AND DEFINITIONS

Machine Learning: A rising area in computer science, where the computer systems are programmed to learn information from rich data sets to produce reliable results to a given problem.

Word Embeddings: A language modeling technique in natural language processing commonly used to represent word tokens into computer-recognizable numeric values by projecting them into a vector space.

Topic Modelling: A frequently used text-mining technique in machine learning and natural language processing which enables us to uncover hidden themes (or topics) in a documents.

Latent Dirichlet Allocation (LDA): A generative topic modelling approach, which assumes that a document is represented as random mixture over hidden themes (or topics). Unlike other topic modelling approaches, LDA can handle unseen documents to discover underlying themes efficiently with a basis of its underlying generative process, which uses the Dirichlet distribution to randomly sample a mixture of hidden themes (or topic).

Dirichlet Distribution: A family of exponential distribution that is commonly used as prior distribution in Bayesian statistics. For example, LDA utilizes the Dirichlet distribution for sampling a mixture of hidden themes.

Perplexity: A measure of how well a sample is predicted by a probability distribution or model. It is often used as a comparison metric in topic modelling.

Automated Essay Scoring: A process of computer program to assign scores to essays in educational assessment.
